# Hybrid umbilical cord blood banking: literature review

**DOI:** 10.1007/s00404-023-07003-x

**Published:** 2023-04-24

**Authors:** Jessica Laue, Johanna Ambühl, Daniel Surbek

**Affiliations:** Department of Obstetrics and Gynecology, University Hospital of Bern, University of Bern, Friedbühlstrasse 19, 3010 Bern, Switzerland

**Keywords:** Umbilical cord blood banking, Public umbilical cord blood banking, Private umbilical cord blood banking, Hybrid umbilical cord blood banking, Hybrid split model, Sequential hybrid model

## Abstract

**Purpose:**

Interest gaps between public and private umbilical cord blood banks have led to the introduction of hybrid banking options. Hybrid models combine features of private and public banks as well as interests of parents, children and of patients, in order to find an optimized solution. While several different models of hybrid banks exist, there is a lack of literature about this novel model of cord blood stem cell banking. Therefore, the aim of this literature review is to assess different options of umbilical cord blood banking and whether hybrid banking could be a valuable alternative to the existing public and private cord blood banking models.

**Methods:**

We performed a systematic literature search, using five main databases. Five hybrid models regarding their advantages as well as their challenges are discussed in this review.

**Results:**

We found that a wealth of literature exists about public cord blood banking, while private and hybrid banking are understudied. Different modalities of hybrid cord blood banking are being described in several publications, providing the basis to assess different advantages and disadvantages as well as practicability.

**Conclusion:**

Hybrid banks, especially the sequential model, seem to have potential as an alternative to the existing banking models worldwide. A previously conducted survey among pregnant women showed a preference for hybrid banking, if such an option was available. Nevertheless, opinions among stakeholders differ and more research is needed to evaluate, if hybrid banking provides the expected benefits.

## Introduction

Umbilical cord blood (UCB) is a source of hematopoietic stem cells (HSC), which has been successfully used as an alternative to bone marrow or mobilized peripheral blood progenitor cells for transplantation. Hematopoietic stem cell transplantation (HSCT) is an important therapeutic option in hematopoietic diseases, such as leukemia as well as other malignant or non-malignant (genetic) conditions [1–4].

There are several advantages of UCB as a source of HSC. Cord blood is widely available, easy to access and the collection is non-invasive, safe and painless. Unlike bone marrow, a higher degree of human leukocyte antigen (HLA) mismatch is tolerated without a higher incidence of graft-versus-host-disease (GVHD) [5, 6].

Since the first UCB transplantation in 1988, more than 40,000 transplants have been performed and multiple studies have shown its potential [2, 7].

The increasing demand for UCB led to the establishment of cord blood banks, where the blood is cryopreserved and stored for future recipients.

The first public bank was established in 1992 [8]. Donated cord blood samples are being HLA-typed and registered in international databases.

As of now, UCB is mostly used for allogeneic (donor is genetically different than the recipient) purpose [3, 7]. Nevertheless, multiple clinical studies showed promising results for autologous (donor and recipient are the same person) treatment of diseases, such as diabetes, cerebrovascular disease or Parkinson’s disease [3, 5]. Even though the promise of regenerative medicine by stem cell treatments is yet to be proven in larger clinical trials, multiple private banks have been established. Private banks offer UCB storage solely for the own child (autologous transplantation) or close family members (allogenic transplantation), raising a storage fee for their service [5]. While HSC transplantation from UCB between HLA-identical siblings is an ideal stem cell option, HLA-haploidentical (“half-identical”, 50% identical) transplantation is becoming widely used if there is no HLA-identical donor, paving the way for an increasing number of UCB transplantations between close family members.

The increasing number of private banks is controversially discussed, as it poses a challenge for public banks to enlarge their inventory, and indications for autologous UCB transplants are currently remote [9].

Given the fact that these tensions may even increase if indications or the demand for autologous grafting expand, an improved solution is needed [1]. To do so, a third banking model, called hybrid banking, was introduced. Some countries have already launched hybrid banks, whereas in Switzerland only public or private banking options are available so far. This initial situation led to the research question of this literature review: “Hybrid umbilical cord blood banking: A new model of cord blood storage?”.

Different hybrid models will be discussed in this review, weighing their advantages and disadvantages, with the aim to assess the possible future role of hybrid banks upon other existing banking options.

## Methods

For this literature review, five databases were mainly searched: Pubmed, Ovid Medline, Scopus, Embase, and Google Scholar. The search strategy included the keywords (umbilical) cord blood bank, umbilical cord blood transplantation (UCBT), hybrid bank, allogeneic-autologous bank, public–private bank, crossover bank, mixed bank, donatable family bank and their combinations. As controlled vocabulary, the MeSH-terms “Cord Blood Stem Cell Transplantation “[Mesh] and “Fetal Blood” [Mesh] AND “Blood Banks” [Mesh] were used.

Additionally, references of articles were looked at to check the integrity of the research and to identify further relevant articles. Results were limited to English articles between 1990 and 2021. Last search was executed on February 12, 2021.

To obtain updated information on cord blood banking in Switzerland, the Swiss Blood Stem Cells (SBSC) from the Swiss Red Cross was contacted via mail.

In total, 327 articles were screened. Among them, 67 were relevant for this review.

## UCB as a source of stem cells

Stem cells are the basic unit of the body. When these divide, they give either rise to new stem cells (self-renewal) or specialized cells with a more specific function (differentiation), such as blood cells, cells of the heart muscle, brain or bone. Totipotent stem cells can differentiate into any cell of the body. This ability to generate new cell types is limited to stem cells, making them an interesting area of research for transplant and regenerative medicine [10]. Umbilical cord blood is a source of multipotent hematopoietic stem cells (HSC), which can primarily differentiate into the three cell lineages of blood cells: erythroid, myeloid and lymphoid [11]. HSC transplantation is used in clinical application to restore the hematopoietic system and/or the immunological function in vivo [3]. HSC have been successfully used to treat diseases, including leukemias, lymphomas, hemoglobinopathies, immunodeficiencies, myelodysplastic and myeloproliferative syndromes and disorders of metabolism [7, 12, 13].

Compared to bone marrow or mobilized peripheral blood progenitor cells, UCB shows several advantages. Besides the easy and safe collection, it can be cryopreserved for years, providing a readily accessible source of stem cells [5, 14]. Furthermore, even without full HLA matching, the incidence of GVHD is low due to the less immunogenic cellular transplants in UCB (T-cell compartment) [7]. Moreover, HLA compatibility is less relevant than with bone marrow transplant (BMT). Additionally, cord blood stem cells have a higher proliferative potential and collection is ethically unproblematic [11].

The main downside of UCB use is the limited cell volume, which may be an obstacle for successful transplantation in adults and adolescents [7]. Moreover, delayed stem cell recovery and higher graft failure was observed [5]. Delayed stem cell recovery describes the issue that compared to bone marrow or peripheral blood transplants, more time is required for the transplanted UCB stem cells to settle in the recipient’s bone marrow and to produce normal blood cells [15].

Methods to improve speed of engraftment and decrease transplant related mortality (TRM) are currently under research. The practice of “double cord” transplantation, using combined cord blood units from two donors, has led to the increasing application of UCBT in adults. Other strategies that are being investigated include in-vitro expansion of cells as well as enhanced homing techniques [5, 14, 16, 17]. When stem cells are infused via intravenous injection, the cells need to migrate and home to the bone marrow. Methods that improve the homing capacity increase the success of cord blood transplants [2].

Besides being a rich source of HSC, UCB has also been found to be a source of non-HSC, such as mesenchymal stromal cells (MSC), T-regulatory cells (Treg), dendritic cells (DC) and natural killer (NK) cells [3]. MSC can differentiate into various tissues including liver, pancreas, neurons, cartilage, bone and muscle, which might play a future role in cell therapy and regenerative medicine. Ongoing research shows promising results for Parkinson’s and Alzheimer’s disease, Huntington, Brain Injury, Cerebral Palsy, Autism, and other conditions [4, 5, 15].

For example, umbilical cord blood derived MSC have shown encouraging results in ameliorating traumatic brain injury (TBI) symptoms in preclinical trials. MSC can promote brain development and may differentiate into neural cell types. Treatment options are currently very limited, therefore autologous or allogeneic stem cell infusions could be a potential therapeutic approach. Advantages of autologous transplantation are that the cells are readily available and the transplanted cells are genetically identical to the recipient, immunosuppressants are thus not required [18, 19].

Furthermore, several randomized controlled trials demonstrated that treatment of cerebral palsy with umbilical cord mesenchymal cells was safe and effective. Results have indicated significant improvements in gross motor and comprehensive functions [20–23]. However, these studies have some limitations. First, numbers of participants were rather small, with heterogeneity of patients within groups. Second, different scoring systems at varied time points were used in the individual trials, making it hard to compare the studies. Taken together, despite showing promising results, more studies that enroll larger numbers of patients and report more standardized outcomes following transplantation are needed before conclusions about efficacy can be drawn [24].

In light of the current pandemic, UCB-derived or cord tissue-derived MSC are under research for treating COVID-19. Apart from the known immunomodulatory effects, MSC have been shown to decrease fibrosis, apoptosis and to induce tissue regeneration, specifically in the lungs. These properties make the use of UC-MSC an interesting therapeutical approach. Preliminary study results are encouraging, but additional research is needed to prove safety and efficiency [25].

## Umbilical cord blood banking

Over the years various models of cord blood banks have been developed. The two most commonly known options are public and private/family banking.

By now, over 40,000 UCB transplantations had been performed worldwide for the treatment of around 80 medical disorders. Approximately 800,000 UCB units are stored in public banks worldwide, while more than 4 million units are stored in private banks [2].

**Fig. 1:**
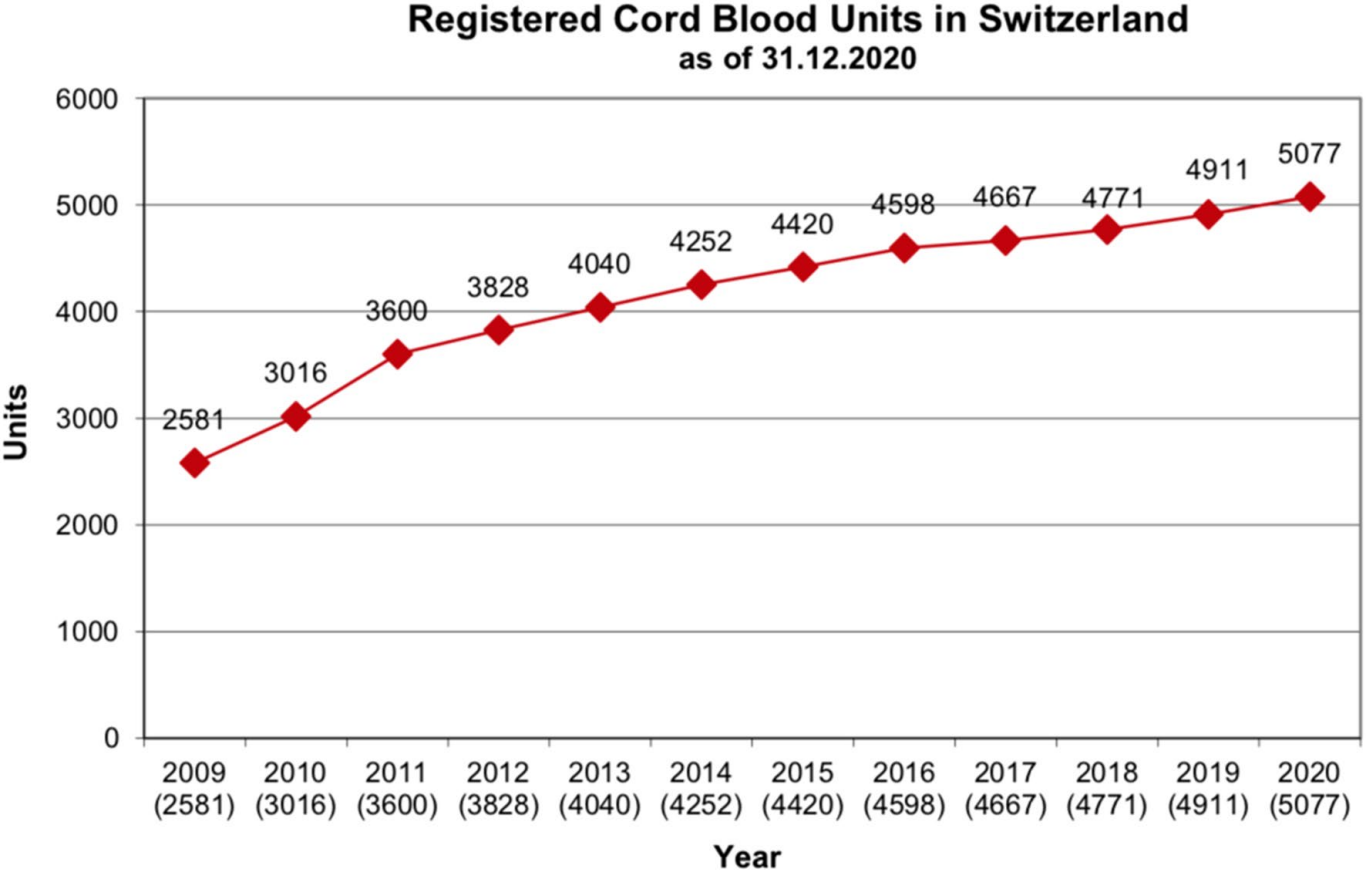
Registered public cord blood units in Switzerland. Source: Swiss Blood Stem Cells (SBSC), Blutspende SRK Schweiz

In Switzerland, one public bank and several private banks are currently running. The public bank is composed of four collection centers (obstetrical clinics of Basel, Bern, Geneva and Aarau), and two storage facilities in Basel and Geneva. The public bank and its centers are coordinated by Swisscord, a commission of the Swiss Red Cross Transfusion organization [26]. By the end of 2020, a total of 5077 UCB units were stored and registered in the public inventory in Switzerland. As shown in Fig. 1, a steady increase has been observed over the last years.

In some countries, including Switzerland, forms of hybrid banking have been developed. Hybrid banks blur the line between the binary of public and private banking, combining aspects of both models.

Most countries apply one or more of the above mentioned cord blood banking options. In the following paragraphs, these models will be discussed (summarized in Table 1).

**Table 1:** Different cord blood banking models and their advantages and disadvantages for customers

Banking model	Advantages	Disadvantages
Public banking	Free of cost for donating parents Inventory can be searched internationally High quality standards Rare HLA types	Funding is needed, lack of financial support, not cost-effective Not available in every country Not available for all women giving birth, only for those in a collection center Many collected UCB units must be discarded before storage because of the high cell number threshold due to economic reasons UCB may not be available for the donor or his family
Private banking	Exclusive access for family members Readily available if need arises Low number of discarded UCB units because of lower cell number threshold	Storage costs paid by parents UCB unavailable to general public Current indications for autologous use of cord blood are low
Hybrid banking		
Sequential hybrid banking	Otherwise privately stored UCB is made available to the public/to patients in need Parents can decide whether to release the cord blood or not	Reconsent from owners is needed before release for unrelated transplant Psychological burden Delay between donor identification and transplantation Storage costs (but reimbursed when donated for unrelated transplant)
Split model	Increases public inventory No reconsent from parents needed Immediate availability	Reduced cell volume Storage costs
Charitable family banking	Money raised by private banking is used for charitable work	
Models promoted by national law	Increase public inventory	Undermining the freedom of choice
Public–private Integration	Financial support of public banks through income from private storage	

### Public banking

Public banks store donated umbilical cord blood for allogeneic use. Costs of collection and storage are covered by the bank, financed through the sale of the cord blood units and supported by the government or foundations [27].

UCB units stored in public banks must meet specific quality criteria, such as a minimum nucleated cell count and volume. In addition to that, collected cord blood needs to be free from microbial contamination and the infant’s birth data must be documented to rule out any communicable diseases. Likewise, a maternal blood sample, as well as the medical and genetic history of the family must be obtained [28]. If quality requirements are not fulfilled, the UCB is discarded or used for research, if parents give consent to do so. According to Guilcher et al., only about 25–40% of the UCB collected meet these criteria [27, 29].

During the storage process, the UCB unit is HLA-typed and the data is put in a registry. This registry can be searched internationally and if a match is found, the cord blood unit will be discharged in exchange of a release fee [30].

There are three prominent organizations for UCB banking in Europe: Eurocord, Netcord/FACT, and JACIE [29].

UCB stored and registered in international stem cell registries often provide stem cells with rare HLA alleles, which can be hardly found in bone marrow repositories. The chance of a sibling being a full HLA match is 25%, and given the small size of modern families, there is a significant proportion of patients (40–50%) for whom neither a sibling donor nor an unrelated bone marrow donor can be found. This is one of the reasons why public cord blood banks were established [11, 31].

Nevertheless, public UCB banks still face an underrepresentation of ethnic minority groups, for which it is consequently difficult to find a suitable donor. This trend is furthermore promoted by the rapid growth of private banks, threatening the supply of cord blood to the public system [31].

By increasing the size of the database, the probability of finding a matching donor is ameliorated. On the other hand, the benefits in terms of a greater number of transplants must be weighed against the additional costs. Querol et al. showed that an inventory of around 1 or 2 units per 1000 population would result in high probabilities of good (5/6 alleles) HLA matches [31].

Indeed, financing of public banks is a challenge. Not every country supports public banks. In Taiwan, for example, the government does not fund umbilical cord blood banks [30]. And even with financial support, UCB storage is very expensive. So sustainable funding may be an issue.

Despite these difficulties, public banking is currently the recommended model by most stakeholders [7, 11, 32–34]. Allogeneic transplant of cord blood has proven to be useful in various diseases, and donation is seen as an act of solidarity for the common good. The greatest disadvantage is that the cord blood might not be available for the donor or his/her family. However, in consideration of the current indications for UCBT, the chance of private cord blood being used is quite low [12]. Estimations range from 1:400 to 1:2500 [35, 36]. Moreover, if the unit has not been released yet, it would still be accessible for personal use. The patient’s family were obligated to pay for the service like everyone else, though [34].

### Private banking

Private cord blood banks are usually for-profit enterprises that offer UCB storage exclusively for the child or family members [29]. For this service, parents generally pay an up-front collection fee, in addition to the annual payments for the ongoing storage [37]. The main benefit of this model is the exclusive accessibility and the immediate availability of the cord blood, should need arise. When public banking is not available, private banking may be the only option to store UCB [27]. For example, Taiwan and India have mostly established private banks because systematic public stem cell banks were neither funded nor promoted by the governments [30, 38]. Furthermore, even in countries such as Switzerland where public banking is well established, only women giving birth in one of the four obstetrical clinics, which are also collection centers of the Swiss UCB bank, can donate UCB at birth. This represents only around 10% of all deliveries in Switzerland, leaving a large gap of almost 90% of women who are not able to donate and store the UCB of their newborn. This inequality is problematic from an ethical point of view.

Also, if the strict inclusion criteria for public banking are not met, private banks still offer parents an opportunity to store their child’s cord blood, as private banking conditions are less restrictive [27]. Specifically, public banks only store UCB with a large nucleated cell number for economic reasons, as the probability of being able to release and sell it increases with a higher cell count.

At the same time, exclusive accessibility is one of the major points of private banking being discussed. By the end of 2013, the inventory of private banks was about 6 times higher than of public banks, yet public banks have released about 30 times more units for transplantation [16].

Today, the principal indication for private banking is direct UCB donation for a sibling with a known disease that can be treated with HSCT. In this case, the healthy sibling’s cord blood is stored exclusively for his/her sick brother or sister. However, most public banks (when available in the area) offer directed family banking too [27].

UCBT is currently in many cases unsuitable for autologous use. In leukemia, for instance, pre-leukemic cells may be already present in fetal blood at birth, before the child develops the full-blown malady [39]. In malignant diseases, allogeneic transplantation has the advantage that the donor’s immune cells can eliminate the patient’s residual malignant cells through the graft-versus-leukemia (GVL) effect. Autologous cord blood can generally not be used to treat a genetic disorder because HSC in the cord blood carry the same genetic defects as the patient [40].

Autologous UCBT shows potential in the field of regenerative medicine. It is hoped that acquired diseases like perinatal brain damage, Parkinson’s or Neuroblastoma can be treated with it. Emerging study results are encouraging, which explains the attractiveness of the private banking market. However, autologous cord blood transplantation is predominantly still experimental and lacks sufficient evidence for general application at this point [33, 41].

In this regard, it seems of high importance that parents are appropriately informed about the advantages and disadvantages of private and public UCB storage before they make a decision [16, 27].

Private banking is associated with significant costs for the family [27], and there is a risk that a bank stops trading or goes bankrupt [42]. Kaimal and colleagues have calculated cost-effectiveness of private banking. They concluded that it is not cost-effective, and that it only becomes economical when banking costs less than $262 or the likelihood of a child needing a stem cell transplant is greater than 1 in 110 [35]. In comparison, public banking is also not cost-effective, if all-over real costs are calculated.

Nevertheless, parents must be informed about the option of private UCB storage and should consider the opportunity to store the cord blood of their child at birth, as research is emerging rapidly and there is no second chance to collect these cells [3].

That said, the American College of Obstetricians and Gynecologists (ACOG) as well as other stakeholders do not recommend private banking for low risk families, since evidence of indication is currently lacking. If an identified family member might need HSCT, private banking should be encouraged [7].

### Hybrid banking

Hybrid banking is a general term for banking practices that combine elements of public and private storage [43]. The common denominator is the aim to make otherwise privately stored UCB units available to the public, thus increasing the diversity and quantity of cord blood for donation. Different models of hybrid banking exist around the world, which will be explained in the following sections.

#### Public–private integration

In this form of hybrid banking, the blood bank runs both; a public and a private sector. The donated blood in the public sector is owned by the bank and can be used for allogeneic transplants as well as clinical trials and research. The privately stored cord blood belongs to the customer, thus can be used for his/her service only. Earnings from the private sector financially support the public storage. This form of hybrid banking is typically used to cope with funding shortfalls, which is particularly eminent when public banking is not supported by the government [30, 44].

One benefit of this hybrid banking model is the active promotion and collaboration with transplantation centers and various stakeholders through the public sector. The extensive network with multiple institutions has led to an increased number of cord blood transplants [30].

Such model of private–public partnership was established in Taiwan [30] as well as in China. In China, public–private integration banks are politically supported by the government. The aim was to cope with funding shortages in an ethically acceptable way [45]. Despite that, it can be observed that cord blood stocked in private banks exceeds the number of publicly stored units in every bank in China. This might be a result of the parents` strong belief that their child’s cord blood could be of personal use in the future. To curb this trend, it has been proposed to define an appropriate ratio of public to private storage for UCB banks [45].

#### Models promoted by national law

In this hybrid model, the government ensures that a part of the private inventory is made available for public healthcare systems, through the enactment of legislation [43].

In Spain, all cord blood units stored privately are recorded in the official Spanish register of Bone Marrow Donors (REDMO). The register can be searched internationally, and if a match is found, the parents are obliged to release the cord blood, whilst the costs for storage are reimbursed [9].

In Turkey, 25% of privately stored cord blood must be offered to the public system. Every year cord blood banks are inspected by the Ministry of Health to check if these regulations are met. This way, the government wants to ensure that public banks have as much units stored as possible [9, 40].

#### Charitable family banking

Charitable family banking is an innovative private banking model, known in Taiwan. The profit from privately stored UCB is used for charitable work such as the free storage of cord blood for high-risk families and ethnic minority groups. The aim is to provide more cord blood units of rare HLA types. This model shows that private banking must not be profit driven, instead it can be used for altruistic purpose [30].

#### Split model

The split model is being offered by the Virgin Health bank in the UK. This hybrid method stores 20% of the UCB unit for private use, whereas the remaining 80% are stored in an inventory available to the public [42, 46, 47].

Advantage of this model is that otherwise privately stored UCB is made accessible to the public, so the inventory of public banks can be increased. Also, the donated 80% are owned by the bank, which means that the cord blood unit is available immediately for transplantation. In addition, Richard Branson, the founder of this model, has warranted that 50% of the profits will be invested in cord blood stem cell research [47]. Since 2018, however, Virgin Health bank closed to new business and changed its name to Precision Cellular Storage. The new business concept focuses on the storage of existing cord blood units only [48–50].

The main downside of the split model is the reduced volume of both cord blood units, so possibly neither part is suitable for transplantation. Proponents of the split model, on the other hand, argue that most likely the donated part would still be accessible, if needed by the donor child or a family member [42].

Apart from that, there is currently ongoing research on in-vitro expansion of stem cells. Two novel compounds, StemRegenin-1 and nicotinamide, have been identified not only to expand cell count and decrease the time of engraftment, but the expanded cells also contributed to long term-engraftment. However, larger phase II and III studies have not been conducted so far [2, 51]. If in-vitro expansion became a standard procedure, the problem of stem cell shortage would be solved [2, 16].

#### Donatable family banking/sequential hybrid banking

The UCB units are primarily collected for family use. At the same time, the stem cells are being HLA-typed and put in a registry. This registry can be searched worldwide and if a match is found, the parents can decide whether they want to release the cord blood unit or not. If they do so, the storage fees will be refunded. Applying this hybrid model, privately stored cord blood may overcome the problem of taking units out of circulation [30, 52]. There are further advantages of this model: all pregnant women have the opportunity for UCB storage and donation, not only those giving birth in a clinic, which is a collection center. Furthermore, UCB units with a nucleated cell number inferior to the required level for public storage can also be stored.

Something to be considered is the volume, quality and accessibility of the units. Private banks do not have the same quality standards as public banks; hence the volume and quality can vary. Also, accessibility is not guaranteed, because consent from the client is needed before release [30].

Besides Taiwan, several banks in Germany have developed this form of hybrid banking. VITA34 offers it as an option called VitaPlusSpende and Eticur calls it Eticur:Kombi [9].

According to VITA34, around 5% of their clients annually opt for the VitaPlus model. Of these units, 20% meet the quality criteria of public banks and have been approved for allogeneic donation [53].

In Switzerland, the commission Swisscord of the Swiss Red Cross recently embarked on a sequential hybrid banking project, together with Swiss Stem Cell Biotech (SSCB) within a public–private-partnership.

## Discussion

Cord blood banks increasingly consider models of hybrid UCB banking, which blur the dichotomy between private and public banking. Different forms of hybrid banks have been introduced around the world, with the intention to respond to current challenges in cord blood banking practices.

In many countries, an organized public banking system is not available. Even in countries such as Switzerland, with a well-established public UCB bank, 90% of women do not have access to public banking. Thus, until today, numerous people only have the choice between private banking or discarding the UCB and letting it go to waste [37]. Even though many women would like to donate their child’s cord blood, the absence of collecting facilities in their living area is frequently a limiting factor [54].

In India and Taiwan, public banking is not supported by the state. Hence, private banks have been established. Hybrid banks seem to be a reasonable alternative for the dichotomous system of public and private banking, combining advantages and overcoming disadvantages of both systems. This may provide opportunities for countries that cannot afford to run public banks. Costs would be covered by private money and the public inventory would be increased. Yet, a requirement for the hybrid banking model is informed decision making with a realistic idea of the medical application of UCB [38].

As in Taiwan, where the release fee for a cord blood unit from a public bank is not covered by health insurance, private banking is the lower-priced option, if a cord blood transplant is actually needed. Even though the likelihood of needing a cord blood transplant is quite small, this is another reason why expecting parents opt for private or hybrid banking [30]. In Taiwan, hybrid banks have released more UCB units for transplantation than public banks. This illustrates that banking options need to be considered in response to different healthcare systems and governance [30].

Development of hybrid banks can also be seen as a response to the threat of state intervention. In Turkey and Spain, it is regulated by law, what proportion of UCB must be released for public donation [9]. In Italy and France, private banking is banned entirely [32, 40]. However, this does not necessarily prevent private banking, as some families choose to store the cord blood in foreign banks [40]. By introducing hybrid models of UCB banks, and thus making privately stored blood available to the public system, private banks try to minimize the need of state intervention [9].

There are some other considerations that will impact the future of hybrid banking models. One factor is the increased application of alternative stem cell sources, such as haploidentical HSCT. Haploidentical HSCT is a newer transplant method, where the donor usually is a close family member with a haploidentical HLA-type. In this procedure, stem cells can be obtained from UCB, from bone marrow or peripheral blood. Transplantation is followed by chemotherapy and high dose cyclophosphamide to reduce the risk of graft failure and GVHD. Haploidentical HSCT is usually less expensive, providing a further advantage of this method [9, 15, 41].

**Fig. 2:**
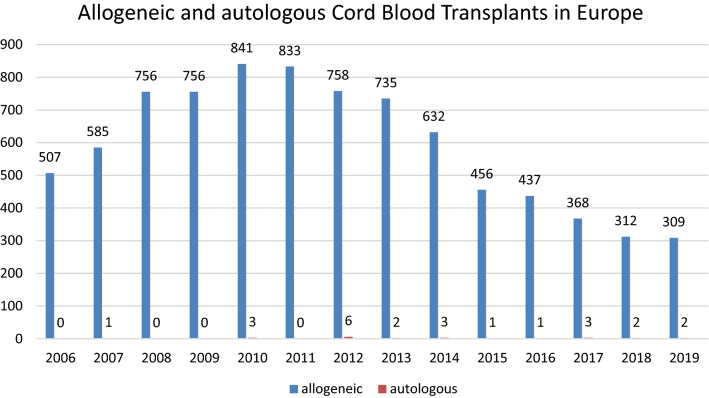
Evolution of cord blood transplants in Europe. Source: European Society for Blood and Marrow Transplantation (EBMT), 2006-2019

**Fig. 3:**
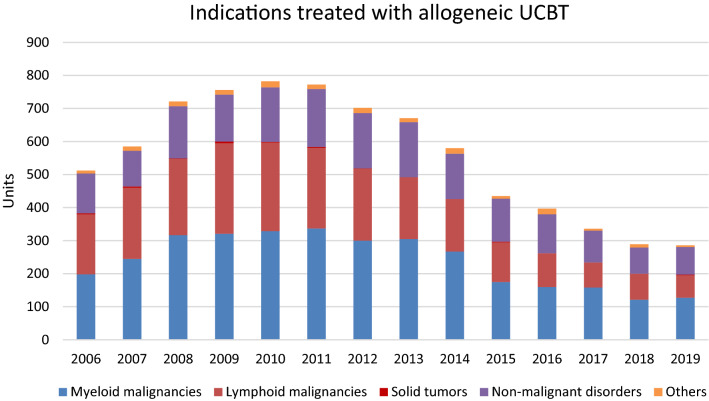
Indications treated with allogeneic UCBT. Source: European Society for Blood and Marrow Transplantation (EBMT), 2006-2019

As shown in Fig. 2, after a steep rise, a decline of UCB transplants has been observed over the last few years in Europe. The number of cord blood transplants performed fell from 841 to 309 between 2010 and 2019. The vast majority of UCBT was used for allogeneic indications, while only zero to six transplants per year were autologous. Conditions treated with autologous UCBT were predominantly non-malignant diseases and solid tumors (Figure not shown) while the main indications for allogeneic transplant were myeloid and lymphoid malignancies (Fig. 3).

Despite the decline of UCBT in the last years, cord blood remains an important treatment modality worldwide, especially for ethnic minority populations. Therefore, cord blood banking should be supported regardless of its current rather infrequent use [15].

The forthcoming role of (autologous) UCB will also depend on the advancement in regenerative medicine [41]. If UCB proves to be beneficial for regenerative therapies, cord blood transplantation is expected to increase sharply [9].

Katz et al. performed a survey where pregnant women from five different countries (Spain, France, Germany, Italy, and UK) were asked about their attitude and knowledge on different cord blood banking options such as public, private and hybrid storage. Among those who opted for storage, more than three quarters stated that they would choose public banking. The main reason for their choice were altruistic thoughts. Only 12% would opt for hybrid banking, as they would not want to miss the one-time chance to store their child’s cord blood, but still consider donating the blood if an unrelated patient is in need [54].

However, 80% of the interviewees declared having poor knowledge of cord blood banking, making it questionable whether an informed decision-making was possible. Moreover, it is not clear if the survey did further specify for which type of hybrid banking the respondents could opt [54].

In addition to that and despite these survey results, it can be observed that the number of privately stored cord blood significantly exceeds the number of publicly stored units [2, 16].

What are the different aspects of hybrid banking discussed above regarding UCB storage in Switzerland? Which form of hybrid banking would be the most applicable?

In Switzerland, a survey study was performed by Wagner et al. They conducted a prospective survey on the acceptance of different cord blood banking options (public, private and hybrid) among actual and potential UCB donors. Results showed that the vast majority accepted UCB collection and would prefer the hybrid banking model, under the condition to be asked for re-consent before the release of UCB for allogeneic use. Two forms of hybrid banking were proposed; split or sequential, whereas participants showed preference for the sequential model. This decision is probably due to the fact that the participants understood that splitting reduces the cell count and thus its utility [52].

A criticism of the sequential model is the psychological burden on the parents when it comes to deciding whether to release the cord blood to an unrelated patient or not. Some argue that this burden is morally unacceptable because human lives may be at stake [40]. This topic was also questioned in the prospective survey by Wagner and colleagues. Interestingly, the majority of participants stated that the psychological burden would only be minor to them [52].

Manegold et al. argue that it might be hard to contact the potential donors for follow-up, due to migration, for instance. Another point is the potential delay between transplant request and release, because re-consent from the donor is needed. For these reasons, some argue that a split hybrid banking model would be more feasible [55]. As mentioned above, the major disadvantage of the split model is the reduced cell count and volume in both portions. Here, the relevance of in-vitro expansion becomes clear.

What should be further mentioned, is the concern that the introduction of a hybrid model as the only type of UCB banking might lead to the loss of a relevant proportion of altruistic donors. A survey conducted upon Swiss public cord blood donors led to the conclusion that almost all donors would choose public banking again, due to altruism and the high costs of private banking. Financial contribution in hybrid banking may discourage public donors from donating because they cannot or do not want to pay for storage [55]. The problem in Switzerland is, however, that 90% of pregnant women in the current public system are deprived from UCB donation. This striking inequality among pregnant women is ethically highly problematic and increasingly difficult to justify.

Another aspect is that financial sustainability is essential for a blood bank to operate. Public banks often struggle to be financially sustainable. Private or hybrid banks, on the other hand, are not confronted with this challenge [15].

Hence, especially if public banking is not possible, hybrid banking is an efficient approach to be economically self-sufficient, while public inventory can be increased [15].

Another subject of importance is the quality standards. Private banks—although they must have high quality standards to be accredited by authorities—usually store UCB with a lower nucleated cell threshold than public banks, as the economic incentive is diametrically opposite. As with all cellular banking for clinical purpose, high quality standards and stringent quality controls are essential for hybrid banks. The searchable inventory should be limited to UCB that meets current public banking accreditation standards [27]. Since UCB units frequently cross international borders, it is generally agreed that universal standards in banking practices are a basic condition for a global UCB operation [29, 37]. In this regard, Ballen and colleagues presume that the introduction of hybrid banking will be beneficial to improve quality of privately used UCB [16].

All things considered, adequate and transparent information about the different banking options is essential. Parents should be able to make an informed decision, based on correct information and unbiased references [38, 56, 57].

Multiple studies have shown that many expecting parents are indeed aware of cord blood banking, but knowledge upon donation possibilities, use of UCB and procedures to be followed is quite low. When asked about the source of information, most interviewees mentioned the internet, media, or information leaflets from hospital clinics, while only a minority of women was informed by their obstetrician or gynecologist [57, 58]. The larger proportion of women stated that they would like to receive more details on the topic and that their antenatal care provider would be their preferred source of information [56]. Providing more details about UCB may result in better knowledge and thus potentially higher donation rates [58].

For that matter, obstetricians and midwives, as well as pediatricians should be educated about UCB banking to be able to discuss this topic with expecting parents [59].

## Conclusion

This literature review aimed to answer the question whether hybrid cord blood banking is a reasonable alternative or addition to the existing banking methods.

Until today, many stakeholders name public banking as the recommended form of cord blood storage. However, they also make clear that recommendations should be reassessed regularly, depending on newly emerging evidence [60]. According to current knowledge, there is only rare indication for autologous use of cord blood, which seems to weaken the justification for private banking. However, with promising ongoing clinical studies, private banks are emerging rapidly, while the existence of public banks is at risk. If not regulated by law, as in Spain and Italy, further UCB might be stored privately and less cord blood units are available to the public. In addition, ongoing funding of public banks seems to be a challenge. As such, hybrid banking might be a good option.

Different forms of hybrid models have been proposed, discussing their advantages and disadvantages.

The split model shows some promising benefits. Public inventory can be increased while part of the cord blood is stored for private use only. However, the success of this model largely depends on the development of in-vitro expansion techniques, which are currently still the subject of research.

The sequential model, on the other hand, provides parents with the opportunity to store their child’s cord blood without being reduced in quantity while they can still decide to release the unit for altruistic donation, if needed.

Hence, a sequential model of hybrid banking seems to be an interesting option in Switzerland. As a prerequisite UCB made available to the public must meet the quality criteria of public banks. In addition, these standards ensure that if used privately, autologous UCBT is of best quality too.

Furthermore, the owners of the cord blood have to be able to be contacted under short notice, to give consent to release the unit in a defined time of period, if a match is found.

All in all, hybrid banking should not be the only banking option. What has become clear, however, is that the simple division between public and private is not adequate anymore.

In summary, it can be concluded that a sequential hybrid banking model has a high potential as an alternative option to the existing banking methods in Switzerland. The ongoing private–public-partnership between Swiss Red Cross and Swiss Stem Cell Biotech is a promising project, eventually leading to the successful introduction of hybrid UCB banking in Switzerland. This model could serve as an example for health care systems around the world.

## Data Availability

Data analyzed in the current research were a re-investigation of existing data, which are openly available in the following databases: Pubmed, Ovid Medline, Scopus, Embase, and Google Scholar.
